# HIV associated factors among men who have sex with men in Maanshan, China: a cross-sectional study

**DOI:** 10.1186/s12981-023-00539-7

**Published:** 2023-07-14

**Authors:** Qi-rong Qin, Ni-ni Qiao, Hong-bin Zhu, Yu-nan Mei, Qian Zhang, Yin-guang Fan

**Affiliations:** 1 Department of AIDS/STD Control and Prevention, Maanshan Center for Disease Control and Prevention, Maanshan, 243011 Anhui China; 2 Department of Epidemiology and Health Statistics, School of Public Health, Anhui Province Key Laboratory of Major Autoimmune Diseases, Anhui Medical University, Hefei, 230032 Anhui China; 3 Global Health Research Center, Duke Kunshan University, Kunshan, 215316 Jiangsu China; 4 Department of Epidemiology and Health Statistics, School of Public Health, Fujian Medical University, Fuzhou, 350122 Fujian China

**Keywords:** Cross-sectional study, HIV/AIDS, MSM, Multivariate analysis, Risk-factors, China

## Abstract

**Background:**

This study conducted a survey of men who have sex with men (MSM) in Maanshan City of Anhui Province to assess the risk behaviors related to human immunodeficiency virus (HIV) infection.

**Methods:**

A cross-sectional survey was conducted from June 2016 to June 2019. The MSM were recruited by a peer-driven sampling method. A face-to-face interview with anonymous questionnaire was used for data collection. The information collected by the survey was summarized and epidemiology described the basic characteristics of MSM, and then the related factors were statistically analyzed.

**Results:**

A total of 934 MSM were recruited with a average age was 30.5 (*SD* = 8.90) years old, including 816 (87.4%) HIV negative participants and 118 (12.6%) HIV positive ones. This study showed that freelancer (*OR* = 4.02, 95% *CI*: 1.96–8.23), scope of sexual partners distribution (*OR* = 1.78, 95% *CI*: 1.36–2.33), number of male sexual partners (*OR* = 2.11, 95% *CI*: 1.47–3.02), role of anal sex with men was receptive (*OR* = 2.54, 95% *CI*: 1.25–5.13) and versatile (*OR* = 2.34, 95% *CI*: 1.31–4.19) and non-steady sex partners (*OR* = 2.14, 95% *CI*: 1.56–2.93) were risk factors for HIV infection, while monthly income (*OR* = 0.68, 95% *CI*: 0.57–0.82), education level (*OR* = 0.79, 95% *CI*: 0.66–0.95), frequency of condom use (*OR* = 0.53, 95% *CI*: 0.35–0.81) and number of oral sex partners (*OR* = 0.35, 95% *CI*: 0.24–0.51) in the past 6 months were protective factors for HIV infection.

**Conclusion:**

Risk behaviors were common in MSM, and urgent need for targeted and comprehensive interventions to reduce risky sexual behaviour and to prevent HIV infection in MSM.

**Supplementary Information:**

The online version contains supplementary material available at 10.1186/s12981-023-00539-7.

## Introduction

Human immunodeficiency virus (HIV) infection and the resulting acquired immune deficiency syndrome (AIDS) is a serious public health threat worldwide. According to the latest global progress report on HIV/AIDS prevention 2021 released by the Joint United Nations Programme on HIV/AIDS (UNAIDS), there was 37.7 million existing HIV/AIDS cases by the end of 2020 and with 1.5 million new infections of HIV/AIDS in 2020 [1], the global spread of the AIDS epidemic is still not promising. Currently, men who have sex with men (MSM) were recognized as high-risk groups for HIV infection [2].

In China, MSM were generally a high-risk population, the proportion of male same-sex sexual transmission increased from 2.5% to 2006 to 28.3% in 2015 among people with HIV/AIDS reported in 2016 [3]. MSM was one of the socially sensitive groups, which easily lead to the transmission of HIV with high-risk sexual behaviors such as multiple sexual partners, frequent changes of sexual partners, high rates of unprotected sex, and complex social networks [4–7]. In addition, more people were looking for sexual partners through the Internet, which not only increases the number of sexual partners, but may also accelerates the spread of HIV [8]. The incidence of new HIV infection was increasing year by year, due to the complexity and secrecy of the sexual behavior relationship network in the MSM, which brings severe challenges to HIV control and prevention work in China [9].

Maanshan City is an inland city of Anhui Province in East China, and although it is located in Anhui Province, it is very close to Nanjing City, the capital of Jiangsu Province. As a central city in the Yangtze River Delta region, Nanjing City has a large number of migrants, and the proportion of HIV positive cases reported in Nanjing increased year by year through sexual transmission, mainly through MSM routes [10]. Influenced by the HIV epidemic in Nanjing City, the HIV epidemic in Maanshan City rose rapidly after the first case of HIV infection was detected in 2002 [11], the total number of HIV infectors was 2.40-fold high in September 2009 compared which in December 2007 [12, 13]. In 2012, the HIV-positive rate was 3.5% among MSM in Maanshan City, which has become one of the most important group for HIV infection [14].

Prior to this, many studies focused on the impact of drug abuse on HIV transmission in Maanshan, but there were no in-depth study on the behavioral characteristics of HIV transmission among MSM [13, 15]. The purpose of this study was to explore the behavioral characteristics of the MSM in Maanshan City, and to compare and analyze the behavior of HIV-positive and HIV-negative in MSM, which was helpful to find some potential influencing factors and has important guiding significance for the HIV control and prevention in MSM.

## Methods

### Study setting

A cross-sectional study was conducted among MSM in Maanshan City from June 2016 to June 2019. All staff underwent rigorous and uniform training prior to the investigation. 30 volunteers were recruited for a pilot survey, the questionnaire (Supplementary material 1) and workflow of investigation were modified according to the feedback of the volunteers. MSM recruited by Blue-Heart working group and a face-to-face interview with anonymous questionnaire was conducted in a separate room upon obtaining informed consent, then, all participants underwent rapid HIV blood testing at the Blue-Heart working group by local Maanshan Center for Disease Control and Prevention (CDC) trained and qualified Blue-Heart working group personnel, with test strips provided by the Maanshan CDC. Early screening HIV-positive participants were referred to laboratory of Maanshan CDC to confirm HIV infection, and obtain the required information from the Integrated HIV Prevention and Control Information System of the Chinese CDC. Fingerprints or unique identification numbers such as WeChat Quick Response (QR) code (contains a specific content format that can only be correctly identification by the WeChat software as a uniqueness for each user) were collected to avoid repetitive investigation. For participants who were not able to complete the survey during the initial visit, a separate appointment was made during a mutually convenient time. Specially-assigned research personnel reviewed the questionnaire on the spot every day, and carried out a supplementary investigation on missing items or logistically promptly.

### Participants

The participants were recruited with a peer-driven sampling method by Blue-Heart working group, a community organization that specialized in HIV test, intervention and counseling services for MSM in Maanshan City. And the inclusion criteria of MSM as follows: (1) aged 18 and above; (2) working, living, or studying more than 3 months in Maanshan City; (3) had anal and/or oral sex behavior with at least one man in the last 6 months; (4) self-reported as HIV negative or unknown. Participants with serious mental illness and refused to write informed were excluded.

### Definition of indicators

1. MSM were defined as cisgender males who self-identified as gay/bisexual or self-reported having anal sex with another male.
2. Multiple partner index defined as having two or more partners who have sex.
3. Indicators of unprotected sex (including oral, anal, and vaginal sex) were defined as anything other than the absence of sex or consistent use of condoms during sex.

### Statistical analysis

Questionnaires were double-entered and then checked for accuracy using EpiData 3.1 (EpiData Association, Odense, Denmark). SPSS version 23.0 (SPSS Inc., Chicago, IL, USA) was used for statistical analysis after logic check and collation. In descriptive analyses, categorical variables were presented as percentage, while continuous variables were expressed as means and standard deviations (*SD*) or median and inter-quartile range (P_25_, P_75_). The Mann-Whitney U test, chi-square tests, chi-square trend test, Poisson regression analysis and multivariate logistic regression analysis model were used for statistical analysis. Poisson regression analysis and multivariate logistic regression analysis models were used to assess the factors influencing HIV infection, and significant variables from the univariate analysis were included in the regression analysis model. *P* < 0.05 was considered to be significant.

### Ethical considerations

The study protocol was approved by the Biomedical Ethics Committee of Anhui Medical University.

Informed consent was asked to sign for all eligible participants when the survey was starting. Participants could receive a gift for prizes of up to 30 Chinese Yuan (CNY) upon the completion of the survey.

## Results

### General demographic characteristics of the MSM participants

As show in Table 1, a total of 934 MSM were investigated in this study, including 816 HIV negative participants and 118 HIV positive, and the overall average age was 30.5 (*SD* = 8.90) years. 73.9% (*n* = 690) participants were Maanshan household registration, and majority of participants (n = 916, 98.1%) were Han ethnic. Most of participants had lower level of education (senior high school or below, n = 687, 73.6%). Besides these, 53.2% (n = 497) participants were unmarried, 75.7% (n = 707) participants’ monthly income over CNY 3 000, and approximate one in third participants were laborer and freelancer.

Table 1 shows that the HIV-positive rates were significantly associated among occupations, education levels and monthly income categories (all *P* < 0.05). The participants of freelancer (n = 58, 20.6%) has highest HIV-positive rate, while the rate of HIV-positive in bachelor degree or above (n = 19, 7.7%) and monthly income less than 1 000 yuan (n = 11, 6.8%) were lowest.

**Table 1:** General demographic characteristics of the MSM^a^ participants in Maanshan, China

Variable		HIV-negative [M(P_25_,P_75_)]/[n (%)]	HIV-positive [M(P_25_,P_75_)]/[n (%)]	*χ*^2^/Z	*P*-value
Total		816(87.4)	118(12.6)		
Age (years old)		30.3(24.0,34.0)	31.8(26.0,36.3)	2.66^b^	0.008
Household registration				1.56^c^	0.458
Maanshan City		601(87.1)	89(12.9)		
Other cities of Anhui Province		122(90.4)	13(9.6)		
Other Provinces		93(85.3)	16(14.7)		
Ethnicity				0.03^c^	0.871
Han		801(87.4)	115(12.6)		
Other nations		15(83.3)	3(16.7)		
Occupation				36.45^c^	< 0.001
Student		146(95.4)	7(4.6)		
Farmer		47(97.9)	1(2.1)		
Laborer		263(88.6)	34(11.4)		
Public institutions		94(93.1)	7(6.9)		
Freelancer		223(79.4)	58(20.6)		
others^d^		43(79.6)	11(20.4)		
Education level				9.31^c^	0.025
Primary school or less		133(85.8)	22(14.2)		
Junior high school		212(83.5)	42(16.5)		
Senior high school/Technical secondary school		243(87.4)	35(12.6)		
Bachelor degree or above		228(92.3)	19(7.7)		
Marital status				3.04^c^	0.219
Unmarried		435(87.5)	62(12.5)		
Married		304(88.6)	39(11.4)		
Divorced/widower		77(81.9)	17(18.1)		
Monthly income (yuan/RMB)				16.21^c^	0.001
< 1 000		151(93.2)	11(6.8)		
1 000–2 999		50(76.9)	15(23.1)		
3 000–4 999		328(84.5)	60(15.5)		
≥ 5 000		287(90.0)	32(10.0)		

^a^ Men who have sex with men; ^b^ Mann-Whitney U test; ^c^ Chi-square test; ^d^ Sex workers, unemployed and retired people

### Sexual orientation and activity range of MSM

Table 2 shows that of the 934 participants, most (n = 722, 77.3%) MSM to find their male partners with Internet or dating Apps, and they had higher HIV positive rate (n = 100, 13.9%), but the difference among age when they first had sex with another man, sexual orientation and main way to find male partners were no significant difference (all *P* > 0.05). From Maanshan City to outside of Anhui Province, HIV positive rates increased with the increased scope of activities (trend *χ*^*2*^ = 39.99, *P* < 0.001), as did the number of MSM friends (trend *χ*^*2*^ = 64.22, *P* < 0.001) and the number of male sexual partners increased (trend *χ*^*2*^ = 37.27, *P* < 0.001).

**Table 2:** Sexual orientation and activity pattern of MSM^a^ in Maanshan, China

Variable		HIV-negative [M(P_25_,P_75_)]/[n (%)]	HIV-positive [M(P_25_,P_75_)]/[n (%)]	*χ*^*2*^Z	*P*-value
Total		816(87.4)	118(12.6)		
Age when they first had sex with another man		22.5(19.0,24.0)	22.0(20.0,23.0)	0.38^b^	0.701
Sexual orientation				-^c^	0.062
Homosexual		795(87.3)	116(12.7)		
Bisexual		4(66.7)	2(33.3)		
Others		17(100.0)	0(0.0)		
Main way to find male partners				4.63^d^	0.201
Bar/dance hall/club		93(91.2)	9(8.8)		
Internet/dating Apps		622(86.1)	100(13.9)		
Public bathhouse		55(90.2)	6(9.8)		
Other ways		46(93.9)	3(6.1)		
Scope of sex partner distribution				41.46^d^ 39.99^e^	< 0.001 < 0.001
In Maanshan City		205(93.2)	15(6.8)		
Cross-region within Anhui Province		318(93.0)	24(7.0)		
Outside of Anhui Province		293(78.8)	79(21.2)		
Number of MSM friends				60.38^d^ 64.22^e^	< 0.001 < 0.001
< 10		86(92.5)	7(7.5)		
11–20		99(92.5)	8(7.5)		
21–50		287(97.0)	9(3.0)		
≥ 51		344(78.5)	94(21.5)		
Number of male sexual partners				31.74^d^ 37.27^e^	< 0.001 < 0.001
< 6		228(97.0)	7(3.0)		
6–10		137(89.5)	16(10.5)		
≥ 11		451(82.6)	95(17.4)		

^a^ Men who have sex with men; ^b^ Mann-Whitney U test; ^c^ Fisher’ exact test; ^d^ Chi-square test; ^e^ Chi-square trend test

### Sexual behavior and condom use among MSM during the past 6 months

Table 3 shows that in the past 6 months, the majority role of anal sex behaviors among participants were versatile, while the HIV positive rate were highest (*χ*^*2*^ = 14.92, *P* = 0.001). HIV positive rate increased with the frequency of condom use decreased from consistent use to never use in anal sex with men (trend *χ*^*2*^ = 44.36, *P* < 0.001). HIV positive rate decreased with the number of oral sex partner increased (trend *χ*^*2*^ = 24.60, *P* < 0.001), in terms of the frequency of condom use in oral sex, the HIV positive rate were highest among the participants who never use condom, but there was no significant difference (*P* = 0.754). 115 (12.3%) MSM ever had group sex, while the HIV positive rate was higher than those who never had group sex (*χ*^*2*^ = 30.65, *P* < 0.001). 31.0% participants had non-steady sex partner with a higher HIV positive rate (20.7%, *χ*^*2*^ = 24.73, *P* < 0.001). Only 3 (0.3%) participants had use illicit drugs in the sex with men, but 2 of them were confirmed HIV positive (*P* = 0.044), and HIV positive rate was higher in participants who self-reported aware of partner’s sexually transmitted diseases (STDs) status before sex (*χ*^*2*^ = 13.49, *P* < 0.001). The results of the univariate logistic regression analysis are consistent with the results of the chi-square analysis, except for age, see Supplementary material 2.

**Table 3:** Sexual behavior and condom use among MSM^a^ in the past 6 months in Maanshan, China

Variable		HIV-negative [n (%)]	HIV-positive [n (%)]	*χ* ^*2*^	*P*-value
Total		816(87.4)	118(12.6)		
Sex role in male anal sex				14.92^b^	0.001
Insertive		179(95.7)	8(4.3)		
Receptive		137(87.3)	20(12.7)		
Versatile		469(85.0)	83(15.0)		
Number of anal sex partner				2.30^b^	0.317
0		31(81.6)	7(18.4)		
1–5		746(87.4)	108(12.6)		
≥ 6		39(92.9)	3(7.1)		
Condom use in anal sex with men				62.43^b^ 44.36^d^	< 0.001 < 0.001
Consistent		37(97.4)	1(2.6)		
Often		90(94.7)	5(5.3)		
Sometimes		379(94.5)	22(5.5)		
Never		279(77.1)	83(22.9)		
Sex role in male oral sex				1.08^b^	0.570
Insertive		22(95.7)	1(4.3)		
Receptive		35(89.7)	4(10.3)		
Versatile		723(87.4)	104(12.6)		
Number of oral sex partner				26.31^b^ 24.60^d^	< 0.001 < 0.001
0		36(80.0)	9(20.0)		
1–2		459(83.5)	91(16.5)		
≥ 3		321(94.7)	18(5.3)		
Condom use in oral sex with men				-^c^	0.754
Consistent		8(100.0)	0(0.0)		
Often		9(100.0)	0(0.0)		
Sometimes		13(92.9)	1(7.1)		
Never		750(87.4)	108(12.6)		
Commercial sex with men (Yes)		50(94.3)	3(5.7)	2.48^b^	0.116
Sexual intercourse with women (Yes)		426(87.8)	59(12.2)	0.20^b^	0.654
Group sex with men (Yes)		82(71.3)	33(28.7)	30.65^b^	< 0.001
Non-steady sex partners (Yes)		230(79.3)	60(20.7)	24.73^b^	< 0.001
Illicit drug use during sex with men (Yes)		1(33.3)	2(66.7)	-^c^	0.044
Aware of partner’s STDs^e^ status (including HIV-negative and HIV-positive status) before sex (Yes)		213(81.0)	50(19.0)	13.49^b^	< 0.001
Using condom when having sex without knowing the STDs status of male partner (Yes)		762(87.0)	114(13.0)	2.81^b^	0.094

^a^ Men who have sex with men; ^b^ Chi square test; ^c^ Fisher’ exact test; ^d^ Chi square trend test; ^e^ Sexually transmitted diseases

### Factors associated with HIV infection in MSM

As show in Table 4, the results of the Poisson model found that the OR increased with scope of sex partner distribution (*OR* = 1.78, 95% *CI*: 1.36–2.33, *P* < 0.001) and number of male sexual partners increased (*OR* = 2.11, 95% *CI*: 1.47–3.02, *P* < 0.001), participants who had group sex (*OR* = 2.53, 95% *CI*: 1.06–5.17, *P* = 0.035) and non-steady sex partners in the past 6 months (*OR* = 2.14, 95% *CI*: 1.56–2.93, *P* < 0.001) had higher risk of HIV infection, meanwhile, the *OR* decreased with monthly income (*OR* = 0.68, 95% *CI*: 0.57–0.82, *P* < 0.001), education level (*OR* = 0.79, 95% *CI*: 0.66–0.95, *P* = 0.011), condom use in anal sex with men (*OR* = 0.53, 95% *CI*: 0.35–0.81, *P* = 0.003) and number of oral sex partner (*OR* = 0.35, 95% *CI*: 0.24–0.51, *P* < 0.001) in the past 6 months increased. Compared to the students, freelancer and other occupation had higher risk to infect HIV (all *P* < 0.05). The participants whose role of anal sex with men was receptive (*OR* = 2.54, 95% *CI*: 1.25–5.13, *P* = 0.010) and versatile (*OR* = 2.34, 95% *CI*: 1.31–4.19, *P* = 0.004) in the past 6 months had higher risk compared to the insertive ones. The results of the multivariate logistic regression analysis are consistent with the results of the Poisson regression model, except for the occupation “laborer”, age, education level and number of MSM friends, see Supplementary material 3.

**Table 4:** Poisson regression analysis of factors associated with HIV infection among MSM^a^ in Maanshan, China

Variable	*β*	SE	Wald	*P*-value	*OR*^*b*^ (95% *CI*^c^)
Age (years old)	0.015	0.010	2.517	0.113	1.02(1.00-1.04)
Education level					
Increase from low level to higher	-0.238	0.090	6.414	0.011	0.79(0.66–0.95)
Occupation (Reference: student)					
Farmer	-0.743	1.111	0.447	0.504	0.48(0.05–4.20)
Laborer	0.683	0.366	3.476	0.062	1.98(0.97–4.06)
Public institutions	0.353	0.523	0.455	0.500	1.42(0.51–3.97)
Freelancer	1.391	0.366	14.479	< 0.001	4.02(1.96–8.23)
Others	1.098	0.414	7.031	0.008	3.00(1.33–6.75)
Monthly income (yuan/RMB)					
Increase from small number to bigger	-0.384	0.092	17.605	< 0.001	0.68(0.57–0.82)
Scope of sex partner distribution					
Increase from small area to bigger	0.575	0.138	17.478	< 0.001	1.78(1.36–2.33)
Number of MSM friends					
Increase from small number to bigger	0.326	0.197	2.744	0.098	1.39(0.94–2.04)
Ever had group sex (Reference: no)	0.853	0.404	4.464	0.035	2.35(1.06–5.17)
The role of anal sex with men in the past 6 months (Reference: insertive)					
Receptive	0.930	0.360	6.693	0.010	2.54(1.25–5.13)
Versatile	0.852	0.297	8.246	0.004	2.34(1.31–4.19)
Condom use in anal sex with men in the past 6 months					
Increase from never use to consistent use	-0.637	0.217	8.648	0.003	0.53(0.35–0.81)
Number of oral sex partner in the past 6 months					
Increase from small number to bigger	-1.061	0.195	29.670	< 0.001	0.35(0.24–0.51)
Non-steady sex partners in the past 6 months (Reference: no)	0.760	0.160	22.447	< 0.001	2.14(1.56–2.93)
Number of male sexual partners					
Increase from small number to bigger	0.744	0.185	16.225	< 0.001	2.11(1.47–3.02)
Illicit drug use during sex with men (Reference: no)	2.123	0.747	8.078	0.004	8.36(1.93–36.14)

^a^ Men who have sex with men; ^b^ Odds ratio; ^c^ Confidence interval; ^d^ Sex workers, unemployed and retired people

## Discussion

This study investigated HIV infection, sexual behavior and associated factors among MSM population in Maanshan City. Previous studies showed that the rate of HIV infection among MSM was 3.5% in Maanshan City in 2012 [14]. The prevalence of HIV was 12.6% (118/934) in this survey, suggesting a marked increase in the HIV prevalence among MSM in Maanshan City in recent years. And the rate of HIV infection in the study was much higher than that of Shandong Province (2.99%) [16] and Fuyang City (5.7%), Anhui Province [17].

In our survey, the average age of participants was 30.5 years and most of the them were young MSM. But the multivariate analysis found that the risk of HIV infection did not gradually increase with increasing age, contrary to findings from other studies showing a higher incidence of HIV in younger MSM [18]. In addition, our study showed that freelancers had higher risk for HIV infection in MSM, which was consistent with finding of Longshuo [19], possibly because of the flexible and less constrained working hours of these occupations.

The present study found that 77.3% of the participants had been found sexual partners through the Internet/dating Apps, which was higher than that (60%) of MSM in Shenyang, China [20]. More and more MSM were looking for sexual partners through the Internet, which may increases the number of sexual partners [8, 20]. Although the number of MSM friends was not an influencing factor for HIV infection in this study, the number of male sex partners and scope of sex partner distribution were risk factors for infection with HIV. The distribution of sexual partners affects the occurrence and transmission range of dangerous behaviors during HIV epidemics, and is a huge challenge for HIV prevention.

About 69.5% of the known HIV transmission routes were associated with sexual behavior in MSM [21]. HIV is mainly transmitted through anal sex among MSM, because it is easy to bleed due to large anal friction during anal sex. Thus, particular modes of sexual behavior increase the risk of HIV infection than in the general population [22]. 99.6% of participants had anal sexual behavior in this survey. What’s more, participants whose role of sex was receptive and versatile were more likely to be infected with HIV than insertive ones, which was confirmed with many findings [23–25]. Besides, 12.3% participants ever had group sex in this survey, and our finding showing a higher positive rate of HIV with ever had group sex. Sex, especially unprotected sex with multiple male partners, was a major risk factor infection with HIV for MSM, and it was important for comprehensive intervention based on risk factors [6, 7]. Furthermore, group sex will increase the risk for HIV infection significantly due to its field specificity and uncontrollability and accelerate the transmission of HIV among MSM [26].

Only 14.8% of MSM consistent used or often used condoms in our study. Unprotected anal sex was a high-risk practice for HIV transmission, and the correct use of condoms can prevent transmission of HIV effectively [27, 28]. The propaganda about correct condom-using among MSM should be strengthened in order to prevent HIV infection themselves in the future. Condom promotion in high-risk population is an effective measure to control and prevent the sexual transmission of HIV, but also an effective means of AIDS prevention and treatment with lower investment and higher income. Besides, intervention efforts targeting MSM should focus not only on the promotion of condom use, but also on changing risky behaviors, in particular, reducing number of non-steady sex partners and their distribution range, and intensive testing of high-risk groups, coupled with the immediate use of antiretroviral treatment (ART) and pre-exposure prophylaxis (PrEP) program, may reduce the prevalence of HIV [29].

### Limitations

There are some limitations in this study. Firstly, this study was a cross-sectional study, which precludes any conclusions on causality. Secondly, the results could only reflect the characteristics and conditions of MSM in Maanshan during the survey, which may not be generalized to the greater population of MSM. Thirdly, drug use was defined as use illegal drugs in this study, such as injction heroin or direct smoking of marijuana, etc. Some new drugs were not on the illegal list such as Rush were not included in our definition of drug use, which may be the main reason why only three participants used illicit drugs. In addition, this study specifically mentioned whether the participants used illicit drugs while having sex with men, rather than in general, which may narrow the scope further.

## Conclusions

The prevalence of HIV in MSM in Maanshan had been increasing rapidly in recent years. Our study confirmed some factors that were associated with an increased risk of sexual transmission of HIV infection in MSM, and reasonable control of the risk factors can effectively reduce the incidence of HIV in MSM.

## Electronic supplementary material

Below is the link to the electronic supplementary material.

**Supplementary Material 1:** Questionnaire.

**Supplementary Material 2:** Table S2 Univariate logistic regression analysis of factors associated with HIV infection among MSM^a^ in Maanshan, China.

**Supplementary Material 3:** Table S3 Multivariate logistic regression analysis of factors associated with HIV infection among MSM^a^ in Maanshan, China.

## Data Availability

The datasets used and/or analyzed during the current study are available from the corresponding author on reasonable request.

## Abbreviations

MSM: Men who have sex with men
HIV: Human immunodeficiency virus
AIDS: Immune deficiency syndrome
UNAIDS: United Nations Programme on HIV/AIDS
CDC: Center for Disease Control and prevention
SD: Standard deviations
STD: Sexually transmitted diseases
ART: Antiretroviral treatment
PrEP: Pre-exposure
χ2: Chi square test
OR: Odds ratio
CI: Confidence interval
