# Supplementary material for: HIV associated factors among men who have sex with men in Maanshan, China: a cross-sectional study
Source: AIDS Res Ther. 2023 Jul 14;20:45. doi: 10.1186/s12981-023-00539-7 (PMC10347791; doi:10.1186/s12981-023-00539-7)
Supplement: Supplementary file 1 — Supplementary Material 1: Questionnaire. [file 12981_2023_539_MOESM1_ESM.doc]

**Questionnaire ID：____________**

**Questionnaire on health and behavior of MSM in Maanshan**

A01 Date of birth Year Month Day

A02 Household registration？

① Our city (Maanshan) ② Other cities of Anhui Province ③ Other Provinces_____

A03 Ethnicity ① Han ethnic ② Other _____

A04 Occupation

① Student ② Farmer ③ Laborer ④ Public institutions ⑤ Freelancer

⑥ Sex workers ⑦ Unemployed ⑧ Retired people ⑨ Other___

A05 Education level

①Primary school or less ② Junior high school ③ Senior high school/Technical secondary school

④ Bachelor degree or above

A06 Marital Status (Marital status with women only):

① Unmarried ② Married ③ Divorced/widower

A07 Your monthly income (yuan/RMB):

① <1000 ② 1000-2999 ③ 3000-4999 ④ ≥5000

B01 What do you consider to be your sexual orientation?

① Homosexual ② Bisexual ③ Others

B02 Your age when you first had sex with another man was _______？

B03 Where/how you most often find male partners?

① Bar/dance hall/club ② Internet/dating Apps ③Public bathhouse ④ Other ways

B04 The scope of sex partner distribution

① In Maanshan City ② Cross-region within Anhui Province ③Outside of Anhui Province

B05 What is the number of your MSM friends?

① <10 ② 11-20 ③ 21-50 ④ ≥51

B06 How many male sexual partners have you had so far?

① <6 ② 6-10 ③ ≥11

C01 How many men have you had anal sex with in the last six months?

①0 (skip to C04) ② 1-5 ③ ≥6

C02 What is your sexual role in male anal sex in the last six months?

①Insertive ② Receptive ③ Versatile

C03 How often did you use condoms during anal sex with men in the last six months?

① Never ② Sometimes ③ Often ④ Consistent

C04 How many men have you had oral sex with in the last six months?

①0 (skip to C07) ② 1-2 ③ ≥3

C05 What is your sexual role in male oral sex in the last six months?

①Insertive ② Receptive ③ Versatile

C06 How often did you use condoms during oral sex with men in the last six months?

① Never ② Sometimes ③ Often ④ Consistent

C07 Have you had commercial sex with a man in the last six months? ①Yes ②No

C08 Have you had sexual intercourse with woman in the last 6 months? ① Yes ② No

C09 Have you had group sex with men in the last 6 months? ①Yes ② No

C10 Have you had a non-steady sex partners in the last 6 months? ①Yes ② No

C11 Have you had sex with a man while using illicit drug in the last 6 months? ①Yes ② No

C12 Do you know the STDs status (including HIV negative and HIV-positive status) of your male partner before you have sexin the last 6 months?

①Yes ② No

C13 Do you use condoms when you have sex without knowing the STD status of your male partner？

① Yes ② No

D01 The results of this rapid HIV test: ①Positive ②Negative

D02 HIV rapid test reagent number：___________

**Investigator: ___________**

**Date of survey: ___________**
