# Supplementary material for: HIV associated factors among men who have sex with men in Maanshan, China: a cross-sectional study
Source: AIDS Res Ther. 2023 Jul 14;20:45. doi: 10.1186/s12981-023-00539-7 (PMC10347791; doi:10.1186/s12981-023-00539-7)
Supplement: Supplementary file 2 — Supplementary Material 2: Table S2 Univariate logistic regression analysis of factors associated with HIV infection among MSMa in Maanshan, China. [file 12981_2023_539_MOESM2_ESM.docx]

**Table S2** Univariate logistic regression analysis of factors associated with HIV infection among MSM^a^ in Maanshan, China

| Variable | *OR*^b^ | *P-value* |  | Variable | *OR*^b^ | *P-value* |
| --- | --- | --- | --- | --- | --- | --- |
| Age (years old) | 1.02 | 0.082 |  | Number of MSM friends |  |  |
| Household registration |  |  |  | <10 | Reference |  |
| Maanshan City | Reference |  |  | 11-20 | 0.99 | 0.989 |
| Other cities of Anhui Province | 0.72 | 0.293 |  | 21-50 | 0.39 | 0.066 |
| Other Provinces | 1.16 | 0.609 |  | ≥51 | 3.36 | 0.003 |
| Ethnicity |  |  |  | Number of male sexual partners |  |  |
| Han | Reference |  |  | <6 | Reference |  |
| Other nations | 1.39 | 0.605 |  | 6-10 | 3.80 | 0.004 |
| Occupation |  |  |  | ≥11 | 6.86 | <0.001 |
| Student | Reference |  |  | Sex role in male anal sex |  |  |
| Farmer | 0.44 | 0.453 |  | Insertive | Reference |  |
| Laborer | 2.70 | 0.020 |  | Receptive | 3.27 | 0.006 |
| Public institutions | 1.55 | 0.424 |  | Versatile | 3.96 | <0.001 |
| Freelancer | 5.43 | <0.001 |  | Number of anal sex partner |  |  |
| othersc | 5.34 | 0.001 |  | 0 | Reference |  |
| Education level |  |  |  | 1-5 | 0.64 | 0.302 |
| Primary school or less | Reference |  |  | ≥6 | 0.34 | 0.141 |
| Junior high school | 1.20 | 0.527 |  | Condom use in anal sex with men |  |  |
| Senior high school/Technical secondary school | 0.87 | 0.636 |  | Consistent | Reference |  |
| Bachelor degree or above | 0.50 | 0.039 |  | Often | 0.20 | <0.001 |
| Marital status(Reference: no) |  |  |  | Sometimes | 0.19 | <0.001 |
| Unmarried | Reference |  |  | Never | 0.09 | 0.019 |
| Married | 0.90 | 0.629 |  | Sex role in male oral sex |  |  |
| Divorced/widower | 1.55 | 0.145 |  | Insertive | Reference |  |
| Monthly income (yuan/RMB) |  |  |  | Receptive | 2.51 | 0.423 |
| <1 000 | Reference |  |  | Versatile | 3.17 | 0.262 |
| 1 000-2 999 | 4.12 | 0.001 |  | Number of oral sex partner |  |  |
| 3 000-4 999 | 2.51 | 0.007 |  | 0 | Reference |  |
| ≥5 000 | 1.53 | 0.242 |  | 1-2 | 0.79 | 0.552 |
| Age when they first had sex with another man (years old) | 0.99 | 0.414 |  | ≥3 | 0.22 | 0.001 |
| Sexual orientation |  |  |  | Condom use in oral sex with men |  |  |
| Homosexual | Reference |  |  | Increase from never use to consistent use | 0.17 | 0.322 |
| Bisexual | 3.43 | 0.158 |  | Commercial sex with men |  |  |
| Others | 0.00 | 0.998 |  | No | Reference |  |
| Main way to find male partners |  |  |  | Yes | 0.40 | 0.128 |
| Bar/dance hall/club | Reference |  |  | Group sex with men |  |  |
| Internet/dating Apps | 1.66 | 0.165 |  | No | Reference |  |
| Public bathhouse | 1.13 | 0.829 |  | Yes | 2.88 | 0.014 |
| Other ways | 0.67 | 0.568 |  | Non-steady sex partners |  |  |
| Scope of sex partner distribution |  |  |  | No | Reference |  |
| In Maanshan City | Reference |  |  | Yes | 2.64 | <0.001 |
| Cross-region within Anhui Province | 1.03 | 0.928 |  | Illicit drug use during sex with men |  |  |
| Outside of Anhui Province | 3.69 | <0.001 |  | No | Reference |  |
| Using condom when having sex without knowing the STDs status of male partner |  |  |  | Yes | 14.05 | 0.031 |
| No | Reference |  |  | Aware of partner's STDsd status (including HIV-negative and HIV-positive status) before sex |  |  |
| Yes | 2.64 | 0.106 |  | No | Reference |  |
| Sexual intercourse with women |  |  |  | Yes | 2.08 | <0.001 |
| No | Reference |  |  |  |  |  |
| Yes | 0.96 | 0.654 |  |  |  |  |

^a^ Men who have sex with men; ^b^ Odds ratio; ^c^ Sex workers, unemployed and retired people; ^d^ Sexually transmitted diseases.
