# Supplementary material for: HIV associated factors among men who have sex with men in Maanshan, China: a cross-sectional study
Source: AIDS Res Ther. 2023 Jul 14;20:45. doi: 10.1186/s12981-023-00539-7 (PMC10347791; doi:10.1186/s12981-023-00539-7)
Supplement: Supplementary file 3 — Supplementary Material 3: Table S3 Multivariate logistic regression analysis of factors associated with HIV infection among MSMa in Maanshan, China. [file 12981_2023_539_MOESM3_ESM.docx]

**Table S3** Multivariate logistic regression analysis of factors associated with HIV infection among MSM^a^ in Maanshan, China

| Variable | β | SE | Wald | *P*-value | *OR^b^* (95% *CI*^c^) |
| --- | --- | --- | --- | --- | --- |
| Age (years old) | 0.041 | 0.018 | 5.103 | 0.024 | 1.04(1.01-1.08) |
| Education level |  |  |  |  |  |
| Increase from low level to higher | -0.329 | 0.168 | 3.835 | 0.050 | 0.72(0.52-1.00) |
| Occupation |  |  |  |  |  |
| Student |  |  |  |  | 1.00 |
| Farmer | -1.373 | 1.722 | 0.635 | 0.425 | 0.25(0.01-7.41) |
| Laborer | 1.099 | 0.646 | 2.895 | 0.089 | 3.00(0.85-10.65) |
| Public institutions | 0.423 | 0.827 | 0.262 | 0.609 | 1.53(0.30-7.73) |
| Freelancer | 2.411 | 0.625 | 14.867 | <0.001 | 11.14(3.27-37.94) |
| Others | 1.710 | 0.801 | 4.558 | 0.033 | 5.53(1.15-26.60) |
| Monthly income (yuan/RMB) |  |  |  |  |  |
| Increase from small number to bigger | -0.708 | 0.183 | 14.947 | <0.001 | 0.49(0.34-0.71) |
| Scope of sex partner distribution |  |  |  |  |  |
| Increase from small area to bigger | 0.945 | 0.219 | 18.595 | <0.001 | 2.57(1.68-3.96) |
| Number of MSM friends |  |  |  |  |  |
| Increase from small number to bigger | 0.622 | 0.251 | 6.140 | 0.013 | 1.86(1.14-3.04) |
| Ever had group sex (Reference: no) | 1.519 | 0.540 | 7.913 | 0.005 | 4.57(1.59-13.17) |
| The role of anal sex with men in the past 6 months |  |  |  |  |  |
| Insertive |  |  |  |  | 1.00 |
| Receptive | 1.630 | 0.563 | 8.391 | 0.004 | 5.11(1.69-15.39) |
| Versatile | 1.464 | 0.496 | 8.709 | 0.003 | 4.32(1.64-11.42) |
| Condom use in anal sex with men in the past 6 months |  |  |  |  |  |
| Increase from never use to consistent use | -1.011 | 0.250 | 16.352 | <0.001 | 0.36(0.22-0.59) |
| Number of oral sex partner in the past 6 months |  |  |  |  |  |
| Increase from small number to bigger | -1.611 | 0.305 | 27.831 | <0.001 | 0.20(0.11-0.36) |
| Non-steady sex partners in the past 6 months (Reference: no) | 1.597 | 0.310 | 26.555 | <0.001 | 4.94(2.69-9.07) |
| Number of male sexual partners |  |  |  |  |  |
| Increase from small number to bigger | 1.068 | 0.293 | 13.266 | <0.001 | 2.91(1.64-5.17) |
| Illicit drug use during sex with men (Reference: no) | 5.857 | 1.804 | 10.547 | 0.001 | 349.77(10.20-11994.94) |

^a^ Men who have sex with men; ^b^ Odds ratio; ^c^ Confidence interval; ^d^ Sex workers, unemployed and retired people.
